# Lysophospholipids: A Potential Drug Candidates for Neurodegenerative Disorders

**DOI:** 10.3390/biomedicines10123126

**Published:** 2022-12-03

**Authors:** Tatsuya Karaki, Hisao Haniu, Yoshikazu Matsuda, Tamotsu Tsukahara

**Affiliations:** 1 Department of Pharmacology and Therapeutic Innovation, Nagasaki University Graduate School of Biomedical Sciences, 1-14 Bunkyo-machi, Nagasaki 852-8521, Japan; 2 Institute for Biomedical Sciences, Interdisciplinary Cluster for Cutting Edge Research, Shinshu University, 3-1-1 Asahi, Nagano 390-8621, Japan; 3 Clinical Pharmacology Educational Center, Nihon Pharmaceutical University, Ina-machi, Saitama 362-0806, Japan

**Keywords:** lysophospholipids, neurodegenerative diseases, amyloid, α-synuclein

## Abstract

Neurodegenerative diseases (NDs) commonly present misfolded and aggregated proteins. Considerable research has been performed to unearth the molecular processes underpinning this pathological aggregation and develop therapeutic strategies targeting NDs. Fibrillary deposits of α-synuclein (α-Syn), a highly conserved and thermostable protein, are a critical feature in the development of NDs such as Alzheimer’s disease (AD), Lewy body disease (LBD), Parkinson’s disease (PD), and multiple system atrophy (MSA). Inhibition of α-Syn aggregation can thus serve as a potential approach for therapeutic intervention. Recently, the degradation of target proteins by small molecules has emerged as a new therapeutic modality, gaining the hotspot in pharmaceutical research. Additionally, interest is growing in the use of food-derived bioactive compounds as intervention agents against NDs via functional foods and dietary supplements. According to reports, dietary bioactive phospholipids may have cognition-enhancing and neuroprotective effects, owing to their abilities to influence cognition and mental health in vivo and in vitro. However, the mechanisms by which lipids may prevent the pathological aggregation of α-Syn warrant further clarification. Here, we review evidence for the potential mechanisms underlying this effect, with a particular focus on how porcine liver decomposition product (PLDP)-derived lysophospholipids (LPLs) may inhibit α-Syn aggregation.

## 1. Overview

Neurodegeneration has been identified as the pathophysiological hallmark in most brain-related disorders. Many neurodegenerative diseases (NDs) involve the misfolding and aggregation of specific proteins into abnormal, toxic species [1,2]. Early diagnosis is essential for treatment planning and ensuring that the right support can be provided to patients and their families. However, therapeutic strategies for NDs present unique challenges for drug development. Although no curative treatment is available for NDs, the range of therapeutic and supportive options are expanding. The most common NDs are Alzheimer’s disease (AD), Lewy body disease (LBD), Parkinson’s disease (PD), and multiple system atrophy (MSA) [3]. The observation of amyloid-like protein aggregation in the brains of patients with NDs was first described in the 1960s, and thus far, three-dimensional structures of several of these aggregates have been elucidated [4,5]. These structural data have been utilized for the design of inhibitors of amyloid-like aggregation proteins [6,7,8]. Since decades, the presence of amyloids in NDs has been associated exclusively with pathologies [9,10]. Additionally, strong evidence indicates that the activation of inflammatory processes is a hallmark of NDs. Elevated levels of pro-inflammatory cytokines and chemokines, as well as activated microglia and astrocytes, are found in the brains of patients with AD, even at very early stages of the disease [11]. Ageing affects homeostatic processes that protect against protein misfolding and is associated with an increase in oxidative stress, neuroinflammation, and mitochondrial-lysosomal dysfunction, which have been shown to directly activate microglia cells and astrocytes [12,13,14,15]. It has been argued that extracellular amyloid-β peptide (Aβ) in AD activates microglia and astrocytes, which in turn release tumor necrosis factor alpha (TNF-α) and other cytokines. TNF-α signaling enhances Aβ production and Aβ-induced neuroinflammation [16]. Neuroinflammation, oxidative stress, and mitochondrial dysfunction have all been linked to the progression of NDs [11,17,18]. The neuroprotective effects of resveratrol reportedly stem from its ability to inhibit microglial activation and regulate neuroinflammation [19,20,21]. Our recent study demonstrated that porcine liver decomposition product (PLDP) could improve cognitive function in elderly adults by providing a rich source of phospholipids (PLs) and lysophospholipids (LPLs) [22]. Notably, the main constituent lipids of synaptic vesicles include cholesterol and phospholipids [23]. Although the concentration of PLDP-derived lipids in LPLs is not sufficient to yield cognitive benefits, LPLs may confer anti-neuroinflammatory effects [24,25]. Previous research suggests that LPLs have served not only as structural components of biological membranes but also as biologically active molecules [26]. LPLs influence a plethora of processes, including neurogenesis in the central nervous system (CNS) [27,28,29]. Growing interest in the involvement of extracellular LPLs in the pathology of NDs increasingly indicates that these small molecules may have therapeutic potential for NDs.

## 2. α-Syn Protein, Aggregates, and Aggregate Inhibitors

### 2.1. α-Syn in Regulating Brain Physiology

In the brain, α-Syn is found mainly in nerve cells in specialized regions called presynaptic terminals [30]. In neurons, α-Syn exists in an equilibrium between cytosolic and membrane-bound states. α-Syn exists as a natively unfolded monomer in the cytosol, whereas membrane-bound α-Syn adopts an α-helical conformation [31]. α-Syn is highly expressed in the neuronal cell bodies of early-stage PD-affected regions, such as the olfactory bulb, dorsal motor nucleus of the vagus, and substantia nigra [32]. Notably, the expression of α-Syn is developmentally regulated. α-Syn mRNA expression begins in late embryonic stages in rodents and peaks in the first few postnatal weeks, following which it is reduced [33]. However, the mechanism by which α-Syn reaches the synapse and its preference for synaptic vesicle membranes remain unclear. Different therapeutic strategies have been developed for reducing brain dysfunction due to protein aggregates, including direct targeting of misfolded proteins [34,35]. NDs are characterized by the misfolding and aggregation of specific proteins [36]. The α-Syn protein is highly expressed in the mammalian nervous system, and the abnormal buildup of α-Syn forms masses known as Lewy bodies [37,38,39,40]. Interestingly, α-Syn oligomers have been found in exosomes, membrane nanovesicles secreted by cells in the CNS [41]. In the CNS, α-Syn is expressed at high levels in neurons of different brain areas such as the neocortex, hippocampus, substantia nigra, thalamus, and cerebellum [42]. The aggregation of α-Syn plays a central role in PD, as well as in other synucleinopathies [43,44]. Abnormal α-Syn yields neuronal cell inclusions and axonal spheroids, as well as oligodendrocyte aggregates (known as glial cytoplasmic inclusions) that accumulate in MSA, rendering α-Syn fibrils important therapeutic targets in PD and related synucleinopathies [45]. PD is the second most common ND, with a global prevalence of over 6 million [46]. Although the exact cause of PD remains unknown, α-Syn has emerged as the major molecule involved in PD pathogenesis. A pathological hallmark of PD is the presence of Lewy bodies, which are intracellular inclusions of aggregated α-Syn [47]. Lewy body dementia (dementia with detectable Lewy bodies) is the third most common types of degenerative dementia. Lewy body dementia causes progressive declines in mental function, and leads to visual hallucinations, movement disorders, cognitive issues, sleep difficulties, attention fluctuations, and depression [48].

### 2.2. Structure of α-Syn

The α-Syn protein, which is 140 amino acids long, comprises three domains: an N-terminal, the non-amyloid-β-component (NAC), and C-terminal domains [49]. All known point mutations in the gene encoding α-Syn (*SNCA*) that are associated with PD are in the N-terminal domain, which is predicted to form an amphipathic α-helix. Additionally, it has a lysine-rich, highly conserved motif similar to that of lipid-binding motifs, and it is responsible for the ability of α-Syn to associate with vesicles and membranes [50,51]. The N-terminal region of α-Syn contains a highly conserved KTKEGV hexamer motif, which yields variations in its surface hydrophobicity [52,53]. Such a periodic motif is characteristic of the amphipathic lipid-binding α-helical domains of apolipoproteins, which have been assigned to subclasses according to their unique structural and functional properties [54,55]. NACore is a peptide fragment spanning residues 68–78 of the α-Syn protein. A previous report suggested that the presence of lipids inhibits NACore (GAVVTGVTAVA) fibril formation. Recently, cryogenic transmission electron microscopy (Cryo-TEM) revealed the presence of non-fibrillar clusters among NACore fibrils formed in the presence of linoleic acid [56]. This effect was additionally observed with PLs and was particularly pronounced with linoleic acid [57]. This inhibitory effect has been attributed to a prolonged lag phase during fibril formation [56]. The α-Syn C-terminal domain contains multiple negatively charged residues, as well as a serine and three tyrosine residues that can be phosphorylated, which may impact α-Syn structure, membrane binding, aggregation, and toxicity [58,59]. The α-Syn protein does not readily aggregate spontaneously [60,61,62], and aggregation is affected by environmental conditions [63,64]. The binding of this protein to lipid membranes offers a possible interface for primary nucleation events to initiate the toxic cascade of α-Syn aggregation. However, previous reports have indicated that α-Syn interacts with certain PLs, which may transform it into a helical conformation [65]. Afitska et al. observed a strong correlation between the induction of α-helix conformation in α-Syn and the inhibition of fibril formation [66]. Thus, helical, membrane-bound α-Syn is unlikely to contribute to aggregation and fibrillation [67]. Small oligomeric forms of α-Syn have been reported to preferentially associate with lipid droplets and cell membranes [68]; α-Syn binds preferentially to small unilamellar vesicles (less than 100 nm in diameter) containing acidic PLs but not to vesicles with a net neutral charge [69]. Nevertheless, according to other reports, α-Syn strongly binds to large unilamellar vesicles with either non-ionic or zwitterionic lipid headgroups [70]. In solution, monomeric α-Syn lacks any secondary structure and specific intrachain interactions and undergoes self-assembly without external additives. However, numerous early epidemiological studies correlated exposure to metals, polyamines, and glycosaminoglycans with α-Syn aggregation [71,72,73,74]. While metal ions can directly cause brain damage, their effect on PD is based on reactive oxygen species (ROS)-dependent effects and a direct influence on α-Syn aggregation [75,76]. The monomeric form of α-Syn is non-toxic, and it does not activate ROS production in neurons [77,78]. In microglia, ROS are generated primarily by NADPH oxidase 2 (NOX2), and activation of NOX2 in disease-associated microglia is associated with damage-associated molecular patterns signaling and inflammation, especially in cerebrovascular diseases [79]. α-Syn-induced activation of microglial NOX2 has been implicated in PD [80]. Activated microglia and accumulation of proinflammatory factors are present in the substantia nigra and striatum of patients with PD [81]. NOX2 is subsequently recognized to be critical for α-Syn-induced microglial activation and neurodegeneration since pharmacological inhibition or gene deletion of NOX2 attenuates α-Syn-induced microglial activation and related neurotoxicity [82,83]. Phagocytosis of α-Syn, with subsequent activation of NOX, plays a central role in the pathogenesis of microglial activation and associated neurotoxicity induced by aggregated α-Syn [14]. α-Syn has been shown to aggregate into various oligomeric and fibrillary forms; soluble, misfolded α-Syn aggregates are neurotoxic and can spread disease via prion-like transfer [84]. α-Syn aggregation is a hallmark of PD, and different forms of the protein can be used for developing quantitative disease models that reproduce the pathological features of this disorder.

### 2.3. Phosphorylation and Ubiquitination of α-Syn

α-Syn is most commonly phosphorylated in serine and tyrosine residues. In Lewy bodies, it is typically phosphorylated at S129 and S87 [62,85,86]. Phosphorylation at S129 is closely linked to PD, increasing from 5% in healthy brains to approximately 90% in Lewy bodies [87,88]. However, the reason behind extensive phosphorylation in the pathology of LBD, such as PD and DLB, is unclear. In vitro studies have offered conflicting conclusions regarding the effect of S129 on α-Syn aggregation [89]. Increased Ca^2+^ influx under mitochondrial impairment has been reported to stimulate a change in the solubility of α-Syn proteins from normally soluble to insoluble and induce Ser129 phosphorylation to generate a proteasomal degradation signal [90]. Because Ser129 phosphorylation plays a role in removing excess amounts of α-Syn, α-Syn aggregates may continuously undergo phosphorylation [89]. It additionally interacts with various proteins, including lipid membranes and fatty acid-binding protein 3, as well as metal ions [75,76,91,92]. In addition, in brain homogenates from diseased human brains and transgenic animals, most S87-P α-Syn was detected in the membrane fractions [93]. S87 is one of the few residues and phosphorylation sites located within the NAC region. A previous study suggested that S87 phosphorylation alters the conformation of membrane-bound α-Syn and decreases its affinity to lipid vesicles, probably by destabilizing the helical conformation and decreasing the lipid-binding affinity of the protein around the phosphorylation site [93]. The cryo-TEM structure of α-Syn fibrils, S87, has been reported to face the outside of the fibril; hence, it remains accessible for disease-associated modification in α-Syn fibrils [45]. Ubiquitination of aggregated or protein filaments has been implicated in the pathogenesis of several NDs [94]. Lewy bodies contain ubiquitin-α-Syn; therefore, they have shown immunoreactivity to anti-ubiquitin antibodies [95]. α-Syn contains eight lysine residues that can be ubiquitinated, and ubiquitin contains multiple internal lysine residues, which can form polyubiquitin chains [96,97]. The presence of ubiquitin in intracellular inclusions in synucleinopathies suggests that abnormally aggregated or misfolded proteins are targeted for ubiquitination in these inclusions in a similar fashion to tau in neurofibrillary tangles of AD [98].

### 2.4. Small Molecule Modulator of α-Syn Aggregation and Drug Development

Approaches for targeting α-Syn monomers have been designed to reduce protein expression, prevent aggregate formation, or promote protein degradation [99]. Studies have shown that α-Syn displays variable kinetics in vitro, and elucidating its fibrillation kinetics would provide valuable information on the molecular events surrounding this process, as well as data required for the screening of small molecule inhibitors against α-Syn aggregation in vitro. Aggregation of α-Syn reportedly affects dopamine metabolism, increases oxidative stress due to mitochondrial dysfunction, disrupts synaptic function, and impairs vesicular trafficking [100]. Major research efforts have been made towards understanding the molecular basis of PD, with the goal of developing therapies for delaying disease progression. Several current therapeutic strategies for PD aim to reduce the neuronal load of aggregated α-Syn. However, the agents used exhibit certain limitations, including degradation by proteases and inefficient crossing of the blood–brain barrier (BBB). Oligomerization inhibitors targeting α-Syn have been widely investigated [101,102,103,104]. For instance, the small molecule UCB0599 (Neuropore Therapies, Inc.) has been shown to cross the BBB with low toxicity in control subjects; it has performed successfully in phase I clinical trials [105]. UCB0599 acts in the first step of the α-Syn aggregation cascade by preventing misfolding and the formation of α-Syn aggregates on lipid membranes [105]. Patients with early-stage PD are currently enrolled in the phase II ORCHESTRA trial for UCB0599, which is expected to finish in October 2023. Small molecule inhibitors are relatively low molecular weight compounds interacting with α-Syn or their aggregation intermediates, altering the amyloidogenic pathway. Treatment approaches using orally available small chemicals appear more suitable for NDs than antibodies and oligonucleotide therapeutics [106], which may potentially have poor pharmacokinetic and pharmacodynamic properties; however, the CNS bioavailability of small molecule therapies requires improvement.

## 3. Effects of LPLs on Cognitive Decline

### 3.1. Dietary Sources of LPLs

Because of their superior emulsification properties, LPLs have numerous applications in the food, cosmetic, and pharmaceutical industries [107]. However, their properties depend strongly on the fatty acid component present and the specific polar head bound to the glycerol backbone [108]. LPLs, as promising feed additives, have been widely used to supplement farm animals diets to improve growth performance, feed efficiency, and dietary fat absorption [109]. LPL supplementation can increase the apparent total tract digestibility of animals [110]. PLs are widely available in the intestinal lumen after eating, and their hydrolysis is catalyzed by phospholipase A2 (PLA2). LPLs (particularly LPC), the digestive products of PLs, have direct roles in mediating chylomicron assembly and secretion [111]. LPC is present in the plasma circulation at relatively high levels and includes species containing both saturated and unsaturated fatty acids [112]. Interestingly, the intake of PL-enriched diets during postnatal brain development was found to increase the number of striatal circuits [113]. In PD, dopaminergic neurons are progressively degenerated, leading to striatal dopamine depletion and movement deficits. α-Syn is intimately involved in the pathogenesis of PD, and has been implicated in the regulation of dopamine synthesis, release, and reuptake [114]. It is possible that dietary PLs have several benefits in health, including improvements in cognition across the lifespan. 

### 3.2. Cognitive Function and LPLs

Mild cognitive impairment (MCI) is characterized by impairment of certain cognitive functions, as defined by Petersen et al. [115], and a diagnosis of mild neurocognitive disorder essentially comprises MCI [116]. It can occur in a subtle form, such as MCI, in the early stages of PD in up to 25% of newly diagnosed patients [117,118,119]. MCI is a distinct stage of cognitive loss that falls between the expected cognitive decline of physiological aging and the more serious loss of mental abilities associated with dementia. It is characterized by impairments in memory, language, thinking, or judgment that are severe enough to interfere with daily life [120]. Individuals with MCI have an increased risk of developing dementia caused by AD or other neurological conditions [121]. Growing evidence supports the hypothesis that dietary factors may play a role in healthy aging, including exertion of a protective effect against age-related cognitive decline [122,123]. The concept of functional foods was first introduced in Japan, a country with a long history of using foods for their health benefits [124]. The market for functional food ingredients covers the sale of functional food ingredients containing bioactive compounds and ingredients used in manufacturing functional food products [125]. The ingredients in functional foods provide health benefits, and some of them include supplements or other additives. Functional foods and bioactive compounds can strongly intensify the therapeutic efficacy of certain drugs by influencing different pathways [126]. A new understanding of these interactions is emerging owing to intense research on nutraceuticals in diseases, including NDs [127]. For example, Yuyama et al. showed that glucosylceramides (GlcCer) from konjac extract are linked to the attenuation of amyloid-like protein in the CNS [128]. In the brains of GlcCer-treated mice, decreased levels of several inflammatory cytokines were accompanied by the recovery of impaired synaptic densities [129]. Future studies need to evaluate GlcCer and examine their relationship with α-Syn in the brain to determine the possible use of plasma GlcCer as a prognostic biomarker of disease progression. Several dietary components including PLs, plasmalogens, ω3 fatty acid, carotenoids, vitamins, and phenolic compounds have been identified to affect cognitive functions [130]. Many studies indicate that neuroinflammation plays a fundamental role in the progression of the neuropathological changes that characterize NDs [131,132,133]; microglia are the main cellular effectors of this process [134]. Indeed, neuroinflammation is initiated by microglia, which are resident immune cells of the CNS. Once activated, microglia can synthesize and release several neurotrophic factors and antioxidants to withstand the pathological progression of NDs [135].

### 3.3. PLDP and LPLs

**Figure 1:**
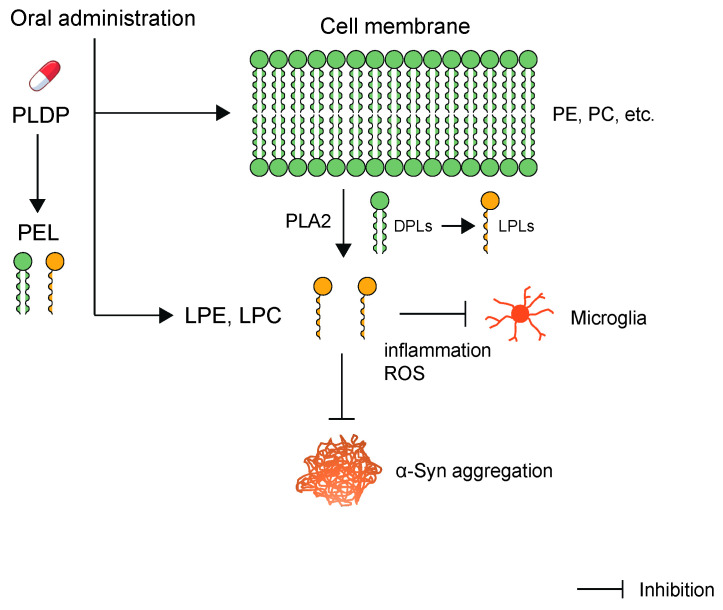
PLDP is a rich source of phospholipids. PLDP extracted lipids (PEL) was extracted from PLDP using the Bligh and Dyer method. This PEL is a rich source of LPLs, including LPC and LPE. LPC and LPE exerted significant protective effects against LPS-induced inflammation and oxidative stress in microglial cells. Various isoforms of PLA2 enzyme hydrolyze PC and PE at the sn-2 position to form LPLs, including LPC and LPE, respectively. α-Syn is bound to LPLs, which are known to be contained in PEL, strongly inhibit α-Syn aggregation.

In previous studies, over 50% of patients with PD without dementia demonstrated cognitive alterations and 20% primarily exhibited memory deficits [136]. We previously reported that PLDP induces a significant increase in the Hasegawa’s dementia scale-revised (HDS-R) score and the Wechsler memory scale (WMS) score in a randomized, double-blind, placebo-controlled study in healthy humans [22]. PLDP was recently approved as a food with functional claims (FFC) in Japan. FFC was introduced in April 2015 for increasing the availability of products that are clearly labeled with certain health functions [137]. Oral administration of PLDP has been shown to enhance visual memory and delayed recall in healthy adults [22]. Notably, the oral route is the most common and preferred method of drug administration for several reasons, such as non-invasiveness, patient compliance, and convenience of drug administration [138]. These results suggest that PLDP may help improve brain function. Interestingly, PLDP is a rich source of PLs, and composition analysis of PLDP revealed that the most abundant PLs and LPLs belonged to the phosphatidylcholine (PC) and phosphatidylethanolamine (PE) classes [25,139]. LPLs influence signaling, proliferation, neural activity, and inflammation to mediate various important processes, including the pathogenesis of cerebral ischemia, vascular dementia, and AD [25,140,141,142]. However, no direct evidence indicates that any PLDP component improves cognitive function, and the effect of PLDP components on cognitive function remains unclear. Our recent study identified novel cooperative actions of LPLs that inhibit IL-6 expression and the accumulation of intracellular ROS in microglia after lipopolysaccharide (LPS)-induced neuroinflammation [25]. This activation was significantly inhibited by lysophosphatidylcholine (LPC) and lysophosphatidylethanolamine (LPE), suggesting that these molecules exert significant protective effects against LPS-induced inflammation (Figure 1). This finding is important considering that microglia-mediated neuroinflammation is regarded as a pathological mechanism in many NDs, such as AD and LBD, and is a pivotal event accelerating cognitive or functional decline. Furthermore, dietary LPC and docosahexaenoic acid (DHA) have been reported to efficiently increase DHA levels in the brain, improving brain function in adult mice [143]. This report describes a novel nutraceutical approach for preventing and treating neurological diseases (such as AD) associated with DHA deficiency. 

## 4. LPLs and NDs

### 4.1. Neuroinflammation and LPLs

PLs are increasingly recognized for their roles in neural function in the brain [144,145,146]. LPLs are glycerophospholipids that lack one acyl chain and possess only one acylated hydroxyl group on the glycerol backbone, rendering them considerably more hydrophilic than diacylglycerol lipids [26,108]. LPLs are found only in small amounts in biological cell membranes in the form of membrane-derived signaling molecules produced by phospholipase A_1_ (PLA1), PLA2, and phospholipase D (PLD) enzymes [108]. The cell membrane comprises phospholipids, with PC and PE being the most abundant [24,139]. PLA_2_ catalyzes the hydrolysis of cell membrane-associated phospholipids to form free fatty acid and LPLs. In cells, these LPLs are intermediate precursors for biosynthesis of other cellular lipids and are thus present at low intracellular concentrations. However, multiple LPLs with varying head groups and hydrocarbon tails are highly abundant in extracellular environments such as the plasma and interstitial fluids [147]. LPLs and their receptors have been detected in a wide range of tissues and cell types, indicating their importance in many physiological processes, including those in the nervous system [148,149]. Our previous study suggested that PLDP, a rich source of LPLs, improves cognitive function in old age [25,139]. LPCs are the major class of LPLs in PLDP, followed by LPE, LPI, and lysophosphatidylserine (LPS). Additionally, traces of lysophosphatidic acid (LPA) have been detected in PLDP [139]. LPLs are amphipathic molecules composed of three sections: a diglycerol group, a hydrophilic head consisting of a charged phosphate moiety, and a small organic molecule covalently bound to the phosphate. The headgroup charge apparently contributes to both strength and specificity in protein interactions. The ability of α-Syn to interact with membrane PLs was recognized after the protein was discovered to be a component of Lewy bodies in the brain [150]. Several studies indicate that membrane interactions promote the folding of α-Syn into amyloid-like structures, which aligns with the direct observation of membranes associated with pathological protein aggregates in many NDs [151]. Furthermore, our recent data showed that the LPS-mediated inflammatory response, including the increase in microglial cytokine production, was suppressed by LPC or LPE exposure. In addition, we observed a synergistic effect between LPC and LPE in LPS-treated microglial cells [25]. LPLs have advantages as CNS-targeting drugs because they are highly lipid-soluble, and they may exhibit good bioavailability. This finding is important considering the role that microglia-mediated neuroinflammation plays in promoting functional decline in many NDs [152].

### 4.2. LPLs and the Brain

PLs and LPLs can transform into each other through the “Lands cycle” to maintain lipid homeostasis [153]. Some lipids, such as LPLs, exhibit neurotransmitter and/or neuromodulatory function, and could act as neuroprotective agents [154,155]. Mouse brain phospholipids have been reported to be highly enriched with long-chain polyunsaturated fatty acids [156,157]. Recent studies show that LPC is the preferred carrier of polyunsaturated fatty acids across the BBB into the brain [158]. The concentration of LPC in the blood plasma of healthy individuals usually ranges from 200 to 300 μM [112]. LPC is produced by the hydrolysis of PC by PLA_2_ (involving the removal of a fatty acid group at the sn-2 position) or by the lecithin cholesterol acyltransferase (LCAT) reaction. In a Drosophila model, PLA2G6 dysfunction, which leads to PARK14-related familial Parkinson’s disease, has been reported to damage the remodeling pathway of phospholipids and induce α-Syn aggregation through alteration of the binding affinity between α-Syn and the synaptic membrane [159]. In circulation, LPC has a short half-life due to rapid degradation, which prevents impairment of various vascular functions [148]. Presently, activated platelets are believed to release LPLs such as LPC, LPE, and LPS, which are then converted to LPA by the lyso-PLD present in the serum [160]. LPC reportedly exerts potent anti-aggregatory effects on platelets [161]. Platelet dysfunction is common during NDs, and impaired platelet function is a feature of several NDs, including AD, PD, Huntington’s disease (HD), amyotrophic lateral sclerosis (ALS), multiple sclerosis (MS), and prion diseases [162]. A recent study confirmed that the modification of lipoproteins by secretory phospholipases inhibited platelet activation and aggregation. The authors identified that LPC plays an essential role in these effects [163]. LPC is increasingly recognized as a key factor positively associated with NDs [164,165], with plasma levels of LPC reported to be decreased in patients with AD [158]. Adults with AD have presented lower LPC concentrations in the blood plasma, cerebrospinal fluid (CSF), and brain tissue than able-bodied individuals [166]. LPC consists of a phosphocholine headgroup and glycerol backbone linked to a variable fatty acid group, bound at either the sn-1 or sn-2 position. In tissues and plasma, polyunsaturated fatty acids (PUFAs) are predominantly bound at the sn-2 position of LPC, whereas saturated fatty acids (SFAs) are typically bound at the sn-1 position [167]. Plasma fatty acids supplied to the brain are derived from two main pools: plasma non-esterified fatty acids and those esterified in the form of LPC. Previous studies have demonstrated that, compared to free fatty acids (FAs), FAs bound to LPCs are more efficiently transported across the BBB into the brain [168,169]. Major facilitator superfamily domain-containing 2A (MFSD2A; currently known as human sodium carbonate electrogenic LPC symporter 1) is an orphan transporter that has been shown to act as a specific receptor for LPC. LPCs transport long-chain PUFAs (such as DHA) across the BBB [170]. Other studies have shown that the brain can only synthesize a few fatty acids; thus, most fatty acids must enter the brain from the blood, passing through the BBB [171]. However, cholesterol and lipoproteins cannot cross the BBB under normal physiological conditions [170,171]. For a small molecule drug to cross the BBB in pharmacologically significant amounts, the molecule must have dual molecular characteristics, namely, a molecular mass of 400–500 Da and high lipid solubility. Therefore, we hypothesize that LPLs may be used for developing an oral, small molecule inhibitor of α-Syn misfolding for slowing disease progression.

### 4.3. LPLs and Therapeutic Potential for NDs

Our recent report proved that LPC16:0, LPC18:0, LPC18:1, and LPE16:0 (known components of PLDP extracted lipids) strongly inhibit α-Syn aggregation [172] (Figure 1). When α-Syn was co-incubated with LPLs, thioflavin-T (an amyloid-binding dye) fluorescent emissions declined remarkably, indicating decreased fibril formation [172]. The role of α-Syn amyloid fibrillation has been recognized in several neurological diseases including PD. In early stages, fibrillation occurs by a transition from a helical structure to extended states in monomeric α-Syn, followed by the formation of β-sheets [173]. This α-helix to β-sheet transition promotes the formation of amyloid fibrils by generating unstable and temporary α-Syn configurations. α-Syn shows variable kinetics in vitro and clarifying its fibrillation kinetics would be valuable for to studying molecular events and screening small molecule inhibitors against α-Syn aggregation [174]. A previous study suggested that several LPLs have a chaperone-like function in protein folding [175]. Monomeric α-Syn is the predominant species in the cytoplasm of cells, and are excreted in cells that do not form a synaptic compartment. However, synaptic membrane binding may be crucial for temporal higher-order multimeric conformation, and PLs appear to have chaperone-like characteristics during this process. These effects could contribute to the neuroprotective effects of LPLs. In the future, we aim to identify functional LPLs contained in PLDP-derived lipids, confirm their clinical effects, and develop new drugs for treating NDs. In particular, we plan to evaluate the efficacy and safety of LPLs using an in vivo model for drug discovery against synucleinopathy. In summary, dietary lipids were originally believed to indirectly affect the brain through their effects on cardiovascular physiology, but are gaining recognition for their direct actions on the nervous system [176]. Further studies are warranted to investigate the role of LPLs in regulating cognition, and the potential for these lipids to reverse age-associated cognitive decline in vivo.

## 5. Conclusions and Future Perspectives

**Figure 2:**
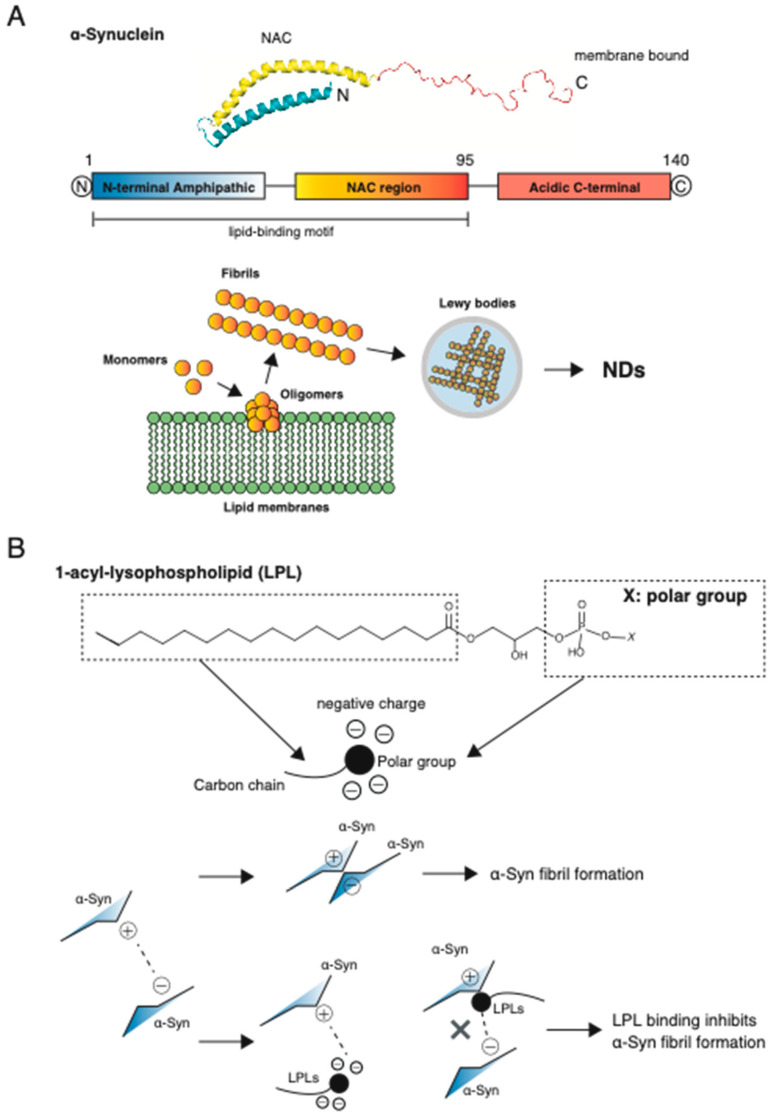
Schematic illustration of the potential mechanism underlying lysophospholipid (LPL) and α-Synuclein (α-Syn) aggregation. (**A**) α-Syn is composed of three domains: the N-terminal, NAC, and C-terminal domains. The N-terminus contains a lipid-binding motif. LPLs lack one fatty acid in comparison to diacylglycerol lipids, and they are much more hydrophilic molecules. (**B**) In aqueous environments, LPLs undergo basic effects, such as the adsorption of the unfolded monomer α-Syn (positive surface charge) upon release from the surface of the cell membrane (negative surface charge). From experimental data, we hypothesize that when α-Syn is bound to LPLs, LPC18:1 and LPE 18:1 (which are known to be contained in PEL) strongly inhibit α-Syn aggregation.

A growing number of publications report that lipids interact with α-Syn during aggregation in NDs. Amyloids are aggregates of associated proteins, and their formation is involved in several diseases often associated with older individuals. The molecular mechanisms influencing the formation of amyloids remain poorly understood. However, amyloid formation in the physiological environment markedly appears in the presence of a large variety of molecules, such as lipids. Unfortunately, no treatment is currently available for curing NDs or altering their progression. However, numerous new treatments are under clinical investigation. Therapeutic drug development requires a deep understanding of the structural properties of α-Syn monomers, oligomers, and fibrils, both in vitro and in vivo. We recently identified functional LPLs within PLDP-derived lipids (PEL) [25]. NDs lack satisfactory treatments primarily because the BBB hinders the passage of drugs from the bloodstream to the brain; this has motivated the development of novel strategies for neuro-drug delivery into the CNS. Thus, drugs need to be modified to improve their delivery. We propose that LPLs are highly attractive research targets in terms of biological activity and possible applications (Figure 2). Additionally, various features of cell membranes (such as lipid composition, charge, curvature, and lipid packing) can modulate the binding of α-Syn to them. We argue that the observed inhibitory effect on fibril formation is due to the association of α-Syn oligomers and LPLs at the early stage of the aggregation process. An important aspect of this mechanism is that it is non-monomeric α-Syn structures that associate with the LPL aggregates. Similar mechanisms of action could be relevant in amyloid formation occurring in vivo, where aggregation occurs in a lipid-rich environment [56]. Under normal conditions, α-Syn adopts an α-helical secondary structure when bound to lipid interfaces, which might be important for explaining differences in how LPLs affect the amyloid formation of different proteins. The ability of LPLs to bind directly to α-Syn and inhibit its aggregation, thereby blocking cell-to-cell propagation of the aggregates, suggests that LPLs may serve as promising therapeutic agents for preventing NDs. Therefore, further studies are necessary for determining the main phospholipids contained within PEL and their effects.

## Abbreviations

α-Syn	α-synuclein
BBB	blood-brain barrier
CNS	central nervous system
DPLs	diacylphospholipids
LPLs	lysophospholipids
PLDP	porcine liver decomposition product
LB	Lewy body
MCI	mild cognitive impairment
PC	phosphatidylcholine
LPA	lysophosphatidic acid
LPE	lysophosphatidylethanolamine
LPC	lysophosphatidylcholine
LPS	lipopolysaccharide
ROS	reactive oxygen species
